# Supplementary material for: A Likelihood Approach for Real-Time Calibration of Stochastic Compartmental Epidemic Models
Source: PLoS Comput Biol. 2017 Jan 17;13(1):e1005257. doi: 10.1371/journal.pcbi.1005257 (PMC5240920; doi:10.1371/journal.pcbi.1005257)
Supplement: S1 File — (TAR.GZ) [file pcbi.1005257.s014.tar.gz › HSPH_Online-SI-Revision/PF08/n100-extreme/n100-extreme_PF_table-4.pdf]

1	R0	{2.76052, 16.3913}	{2.17419, 16.6409}	{{2.16851, 16.4944}, {2.17926, 16.8436}}	{21.2397, 1.5227}	inf	10 419.	6559.8	{6486.34, 6654.43}	37.04	0.200957	newly	5804.	3339.25	{3262., 3418.}	42.4664	0.240138	Reff	2.28524	1.91987	{1.91479, 1.92484}	15.9882	ar	92 090.	87 423.2	{86 950., 87 799.}	5.06765
2	R0	{2.18202, 15.1734}	{2.19673, 20.}	{{2.18008, 20.}, {2.20004, 20.}}	{0.82882, 31.8096}	inf	10 970.	11 890.3	{11 142.9, 12 208.}	8.60947	0.035667	newly	5914.	4689.57	{4380., 4845.}	20.7039	0.101063	Reff	1.6841	1.73136	{1.72095, 1.74006}	2.8059	ar	84 264.	93 592.9	{92 850., 94 612.}	11.071
3	R0	{2.57891, 12.5971}	{2.91004, 20.}	{{2.86463, 20.}, {2.95033, 20.}}	{12.8398, 58.7667}	inf	8525.	11 005.5	{10 545., 11 407.1}	29.0971	0.110676	newly	6571.	4914.56	{4695., 5093.}	25.2083	0.12638	Reff	2.202	2.44387	{2.39988, 2.48176}	10.984	ar	90 302.	97 572.8	{97 148., 98 150.}	8.0516
4	R0	{2.5856, 9.28508}	{2.9399, 17.0871}	{{2.85146, 14.999}, {3., 20.}}	{14.2752, 84.027}	inf	2564.	3464.72	{2931., 3956.14}	35.457	0.129413	newly	3492.	2108.27	{1576., 2822.}	39.6257	0.230787	Reff	2.47933	2.7237	{2.47764, 2.84057}	10.6519	ar	90 312.	95 448.7	{94 177., 96 755.}	5.814
5	R0	{2.4406, 12.1341}	{2.829, 20.}	{{2.77145, 20.}, {2.87396, 20.}}	{15.9142, 64.8248}	inf	4472.	6178.45	{5783.66, 6574.97}	38.1586	0.139818	newly	3644.	2859.14	{2662., 3061.}	21.5384	0.105945	Reff	2.2517	2.58799	{2.53305, 2.62864}	14.935	ar	88 597.	96 736.2	{95 997., 97 381.}	9.18677
6	R0	{2.72155, 15.4094}	{2.17212, 16.7732}	{{2.16443, 16.6791}, {2.1777, 16.854}}	{20.1881, 8.85044}	inf	8392.	5586.84	{5549.78, 5629.59}	33.4265	0.176735	newly	5118.	2854.92	{2793., 2929.}	44.2181	0.253635	Reff	2.34317	1.96821	{1.96117, 1.9721}	16.0025	ar	91 682.	88 456.8	{88 165., 88 800.}	3.51782
7	R0	{2.63781, 14.3935}	{2.12093, 20.}	{{2.10868, 20.}, {2.1297, 20.}}	{19.5952, 38.9516}	inf	9079.	5777.75	{5724.94, 5816.02}	36.3614	0.196288	newly	5875.	2374.58	{2313., 2431.}	59.5816	0.393511	Reff	2.22919	1.93014	{1.91992, 1.93824}	13.4153	ar	91 192.	89 101.3	{88 637., 89 556.}	2.29261
8	R0	{2.82391, 16.104}	{2.13303, 16.6565}	{{2.12138, 16.6122}, {2.14235, 16.71}}	{24.4653, 3.43093}	inf	8948.	5727.29	{5677.44, 5795.78}	35.9936	0.193837	newly	5170.	2917.49	{2820., 2991.}	43.5689	0.248586	Reff	2.41478	1.92068	{1.91058, 1.92766}	20.4616	ar	92 664.	86 905.6	{86 459., 87 367.}	6.21427
9	R0	{2.80645, 14.6457}	{2.13104, 17.4115}	{{2.12434, 17.2108}, {2.14056, 17.6685}}	{24.0663, 18.8847}	inf	5809.	4692.09	{4571.28, 4753.31}	19.2273	0.0928449	newly	4055.	2291.17	{2154., 2380.}	43.4977	0.248274	Reff	2.54301	1.97319	{1.96477, 1.985}	22.4073	ar	92 567.	88 210.9	{87 853., 88 498.}	4.70592
10	R0	{1.86213, 5.4271}	{2.99866, 19.8676}	{{3., 20.}, {3., 20.}}	{61.034, 266.081}	inf	1977.	5808.34	{5358.04, 6232.66}	193.795	0.467314	newly	4393.	2790.1	{2648., 3002.}	36.4876	0.197665	Reff	1.77612	2.73547	{2.72661, 2.77614}	54.0142	ar	75 362.	97 695.1	{96 877., 98 326.}	29.6345
11	R0	{2.27349, 14.6338}	{2.22118, 17.7469}	{{2.21445, 17.5699}, {2.22606, 17.8497}}	{2.31572, 21.2732}	inf	10 491.	10 435.9	{10 366., 10 508.}	0.66891	0.00292885	newly	6044.	4800.42	{4712., 4899.}	20.5754	0.100135	Reff	1.80677	1.82339	{1.81789, 1.82762}	0.920315	ar	85 805.	92 383.9	{92 016., 92 720.}	7.6673
12	R0	{2.77261, 14.1894}	{2.17907, 19.703}	{{2.02211, 17.1719}, {2.20938, 20.}}	{21.4072, 38.8574}	inf	6492.	5006.05	{4287.46, 5160.43}	22.889	0.113537	newly	4508.	2123.18	{2049., 2193.}	52.902	0.327158	Reff	2.48165	2.01253	{1.88492, 2.03783}	18.9037	ar	92 508.	89 474.3	{86 454., 90 408.}	3.27941
13	R0	{2.65402, 19.3935}	{2.19137, 20.}	{{2.1829, 20.}, {2.19756, 20.}}	{17.4319, 3.12734}	inf	11 250.	6955.81	{6899.47, 7013.6}	38.1706	0.20882	newly	4926.	2799.17	{2728., 2860.}	43.1756	0.245552	Reff	2.14402	1.81878	{1.81015, 1.82571}	15.1699	ar	91 184.	91 354.4	{90 829., 91 838.}	0.392668
14	R0	{1.75297, 4.45354}	{2.0826, 19.0125}	{{1.85406, 16.6185}, {2.18595, 20.}}	{19.8078, 326.908}	inf	699.	1651.39	{1216.18, 1997.43}	136.251	0.365825	newly	1855.	736.22	{590., 881.}	60.3116	0.408178	Reff	1.72278	2.00524	{1.8364, 2.11638}	17.3632	ar	71 263.	85 297.	{79 724., 88 070.}	20.1598
15	R0	{1.99928, 7.1501}	{2.86758, 20.}	{{2.81045, 20.}, {2.90024, 20.}}	{43.4304, 179.716}	inf	3389.	7091.68	{6663.74, 7560.79}	109.256	0.320258	newly	4922.	3266.06	{3038., 3465.}	33.6436	0.178583	Reff	1.85853	2.5771	{2.53229, 2.6129}	38.6635	ar	79 602.	97 139.8	{95 892., 98 394.}	22.0319
16	R0	{2.67398, 10.3134}	{2.80544, 20.}	{{2.79043, 20.}, {2.81318, 20.}}	{4.91644, 93.9225}	inf	4133.	5303.19	{5233.53, 5339.08}	28.3134	0.108253	newly	4823.	2464.9	{2392., 2527.}	48.8928	0.291608	Reff	2.49563	2.60693	{2.59124, 2.61502}	4.46	ar	91 289.	96 775.8	{96 361., 97 092.}	6.01038
17	R0	{2.44634, 16.3226}	{2.21473, 20.}	{{2.20629, 20.}, {2.22865, 20.}}	{9.46775, 22.5295}	inf	10 530.	10 211.2	{9841.87, 10 390.}	3.0275	0.0134355	newly	5419.	4118.	{3973., 4234.}	24.0081	0.11935	Reff	1.9738	1.85301	{1.84104, 1.87388}	6.12002	ar	88 710.	94 217.6	{93 813., 94 575.}	6.20854
18	R0	{2.02168, 10.1676}	{2.11085, 20.}	{{2.10764, 20.}, {2.11297, 20.}}	{4.41093, 96.7033}	inf	4597.	4647.75	{4631.77, 4665.21}	1.14471	0.00494345	newly	4153.	1872.44	{1822., 1918.}	54.9136	0.346041	Reff	1.82331	1.82577	{1.82242, 1.82792}	0.154614	ar	80 299.	87 248.5	{86 867., 87 653.}	8.65455
19	R0	{2.89969, 15.009}	{2.18041, 17.4977}	{{2.1814, 17.5091}, {2.1814, 17.5091}}	{24.8053, 16.5814}	inf	6390.	4908.44	{4918.17, 4918.17}	23.1856	0.114603	newly	4254.	2407.6	{2343., 2467.}	43.4039	0.24731	Reff	2.60468	2.01546	{2.01611, 2.01611}	22.6215	ar	93 349.	89 264.3	{88 946., 89 594.}	4.37575
20	R0	{2.68933, 7.34928}	{2.0295, 17.834}	{{1.63352, 14.1751}, {2.73105, 20.}}	{25.1649, 142.664}	inf	478.	656.129	{389.671, 1114.3}	45.1972	0.143767	newly	1059.	321.79	{187., 536.}	69.6138	0.55775	Reff	2.66728	2.00406	{1.60332, 2.69252}	25.4254	ar	91 574.	80 678.9	{69 490., 94 989.}	13.3687
21	R0	{2.15352, 9.28074}	{2.88002, 20.}	{{2.81551, 20.}, {2.92715, 20.}}	{33.7353, 115.5}	inf	3961.	6886.67	{6581.49, 7353.32}	73.8619	0.239854	newly	4282.	3190.9	{3037., 3387.}	25.4811	0.128098	Reff	1.99267	2.59949	{2.54288, 2.63504}	30.4523	ar	83 845.	97 227.5	{96 373., 98 401.}	15.961
22	R0	{2.21953, 9.04387}	{3., 20.}	{{3., 20.}, {3., 20.}}	{35.1638, 121.144}	inf	3707.	6851.16	{6523.99, 7083.3}	84.8168	0.266516	newly	4322.	3235.42	{3097., 3361.}	25.1407	0.12597	Reff	2.06581	2.70917	{2.69715, 2.72267}	31.1433	ar	84 856.	96 684.4	{96 363., 96 916.}	13.9394
23	R0	{2.21987, 14.5347}	{1.95126, 20.}	{{1.95003, 20.}, {1.95296, 20.}}	{12.1001, 37.6017}	inf	10 313.	6580.46	{6553., 6610.96}	36.1926	0.195138	newly	5905.	2602.58	{2550., 2664.}	55.9258	0.355892	Reff	1.76493	1.70128	{1.70019, 1.70259}	3.60613	ar	85 067.	83 893.3	{83 517., 84 288.}	1.37969
24	R0	{2.84268, 17.9659}	{1.94837, 20.}	{{1.94188, 20.}, {1.95369, 20.}}	{31.46, 11.322}	inf	10 277.	6013.66	{5956.65, 6065.82}	41.4843	0.232754	newly	5220.	2381.6	{2321., 2439.}	54.3755	0.340881	Reff	2.35894	1.71589	{1.71151, 1.71944}	27.2603	ar	92 836.	83 322.4	{82 819., 83 788.}	10.2478
25	R0	{2.52706, 10.3679}	{2.06838, 20.}	{{2.06724, 20.}, {2.06921, 20.}}	{18.1506, 92.9031}	inf	4409.	3854.27	{3846.38, 3859.98}	12.5817	0.0583984	newly	4655.	1588.	{1544., 1633.}	65.8861	0.467169	Reff	2.33579	1.93451	{1.93309, 1.93648}	17.1794	ar	89 623.	85 677.1	{85 337., 86 077.}	4.40279
26	R0	{2.62835, 19.1088}	{2.26997, 20.}	{{2.26074, 20.}, {2.27906, 20.}}	{13.6353, 4.66382}	inf	12 549.	11 233.8	{10 952.7, 11 422.}	10.4803	0.048216	newly	5505.	4540.06	{4394., 4658.}	17.5284	0.0838657	Reff	2.05547	1.86547	{1.85689, 1.87549}	9.24378	ar	90 908.	95 699.8	{95 248., 96 122.}	5.27108
27	R0	{2.76831, 19.4155}	{2.15346, 17.0128}	{{2.13621, 16.8145}, {2.19133, 17.5905}}	{22.2102, 12.375}	inf	16 382.	10 448.2	{10 226.3, 10 695.9}	36.2212	0.195373	newly	6900.	4954.95	{4851., 5057.}	28.1891	0.143873	Reff	1.96727	1.72981	{1.7211, 1.75856}	12.0705	ar	92 175.	89 152.6	{88 729., 89 824.}	3.27901
28	R0	{2.96609, 16.8903}	{2.99883, 20.}	{{3., 20.}, {3., 20.}}	{1.1238, 18.4112}	inf	11 248.	12 103.3	{10 947.6, 12 706.5}	8.69108	0.0360055	newly	6075.	5425.87	{4925., 5717.}	10.6853	0.0496171	Reff	2.44352	2.48078	{2.44944, 2.53576}	1.53294	ar	94 024.	98 442.6	{98 081., 98 752.}	4.6994
29	R0	{2.17046, 14.39}	{1.97878, 20.}	{{1.97377, 20.}, {1.98296, 20.}}	{8.83146, 38.9854}	inf	8850.	6444.47	{6361.78, 6497.2}	27.1811	0.137774	newly	5160.	2568.99	{2503., 2637.}	50.2134	0.302961	Reff	1.78813	1.734	{1.72945, 1.73828}	3.02711	ar	84 001.	85 215.5	{84 766., 85 563.}	1.44579
30	R0	{2.88772, 12.4647}	{3., 16.1332}	{{3., 15.7336}, {3., 16.448}}	{3.88819, 29.4315}	inf	3959.	4344.7	{4136.86, 4537.91}	9.78581	0.0402041	newly	3670.	2841.71	{2628., 3051.}	22.5692	0.11203	Reff	2.71599	2.83769	{2.82773, 2.84579}	4.4809	ar	93 273.	97 386.7	{97 001., 97 731.}	4.41042
31	R0	{2.46831, 8.14308}	{2.72705, 15.7384}	{{2.11753, 13.651}, {3., 20.}}	{18.5167, 93.2732}	inf	1290.	1837.08	{1192.21, 2463.45}	47.711	0.163683	newly	2181.	1212.64	{764., 1737.}	44.3998	0.27807	Reff	2.41433	2.61365	{2.05686, 2.92331}	16.3181	ar	88 762.	92 605.5	{84 747., 96 682.}	7.18305
32	R0	{1.93236, 7.38987}	{3., 20.}	{{3., 20.}, {3., 20.}}	{55.2506, 170.641}	inf	2899.	7292.59	{7048.02, 7533.96}	151.555	0.400438	newly	4137.	3429.83	{3308., 3558.}	17.0938	0.0816455	Reff	1.80908	2.70423	{2.69342, 2.7151}	49.4811	ar	77 654.	97 725.1	{97 452., 97 980.}	25.8469
33	R0	{2.82251, 17.6954}	{2.83795, 19.9067}	{{2.80661, 20.}, {2.88834, 20.}}	{1.48274, 13.2907}	inf	8313.	8933.08	{8442.41, 9574.74}	8.50431	0.0359408	newly	4307.	4046.05	{3810., 4320.}	6.52914	0.0297166	Reff	2.44362	2.47904	{2.46683, 2.51113}	2.12907	ar	92 745.	97 042.2	{96 719., 97 665.}	4.94266
34	R0	{1.87083, 12.3994}	{2.24253, 20.}	{{2.22155, 20.}, {2.26098, 20.}}	{19.8684, 61.2981}	inf	6523.	9153.33	{8994.35, 9319.7}	40.3239	0.147065	newly	4361.	3657.58	{3556., 3759.}	16.1298	0.0765061	Reff	1.57679	1.7728	{1.75845, 1.78614}	12.4308	ar	76 282.	93 749.1	{93 137., 94 327.}	22.8981
35	R0	{2.95779, 16.3003}	{2.14667, 16.1316}	{{2.11149, 16.1544}, {2.1625, 16.1544}}	{27.4231, 1.34211}	inf	6128.	4156.28	{3857.45, 4248.14}	32.1756	0.169587	newly	3852.	2237.48	{2030., 2340.}	41.9138	0.236688	Reff	2.67834	1.99566	{1.97104, 2.00782}	25.4888	ar	93 873.	86 974.3	{85 827., 87 710.}	7.349
36	R0	{2.18718, 14.261}	{2.16843, 16.3516}	{{2.14939, 16.0405}, {2.18102, 16.8454}}	{0.860392, 14.6596}	inf	9128.	9229.14	{9106.32, 9348.9}	1.35986	0.0058615	newly	5307.	4655.92	{4484., 4801.}	12.2683	0.0570304	Reff	1.78461	1.80251	{1.78719, 1.8139}	1.03985	ar	84 379.	88 066.5	{87 644., 88 513.}	4.37018
37	R0	{2.44912, 9.31118}	{2.99958, 20.}	{{3., 20.}, {3., 20.}}	{22.476, 114.796}	inf	3260.	5755.02	{5481.23, 6203.28}	76.5343	0.246229	newly	3995.	2742.39	{2582., 2936.}	31.3544	0.163927	Reff	2.30876	2.75126	{2.72134, 2.77576}	19.1661	ar	88 442.	97 281.8	{96 811., 97 751.}	9.99506
38	R0	{2.05949, 5.72514}	{2.55079, 19.2354}	{{2.07479, 16.4661}, {2.79387, 20.}}	{26.4251, 235.98}	inf	809.	1818.11	{1010.37, 2208.69}	125.893	0.340714	newly	2064.	866.18	{572., 1032.}	58.0339	0.388608	Reff	2.02491	2.4883	{2.04715, 2.70717}	25.6213	ar	81 587.	91 967.2	{84 496., 96 178.}	14.5881
39	R0	{1.75505, 8.52404}	{2.28623, 19.8638}	{{2.21234, 20.}, {2.3299, 20.}}	{30.266, 133.033}	inf	4468.	8702.4	{7854.71, 9110.52}	94.7717	0.288836	newly	4543.	3632.07	{3367., 3817.}	20.0513	0.0977751	Reff	1.55847	1.99357	{1.91852, 2.02724}	27.9187	ar	70 925.	95 479.1	{94 351., 96 327.}	34.6198
40	R0	{2.24724, 13.7861}	{2.44092, 18.231}	{{2.1906, 16.7138}, {2.75656, 20.}}	{10.9094, 32.2418}	inf	10 101.	13 105.3	{9830.53, 17 176.5}	31.3099	0.104306	newly	6145.	5815.44	{4814., 7051.}	17.5873	0.0806999	Reff	1.78404	1.89894	{1.81215, 2.00018}	6.44042	ar	85 257.	93 025.5	{89 482., 97 324.}	9.11187
41	R0	{1.77635, 8.62406}	{2.41592, 17.7261}	{{1.99695, 13.0459}, {2.70473, 20.}}	{36.0049, 105.542}	inf	5330.	10 818.1	{7639.56, 13 018.}	102.967	0.297238	newly	5334.	5158.53	{4775., 5560.}	5.60161	0.0251598	Reff	1.53452	2.0202	{1.74468, 2.21111}	31.6506	ar	72 941.	94 680.9	{87 740., 98 658.}	29.8047
42	R0	{1.80397, 5.4932}	{2.99914, 19.8605}	{{3., 20.}, {3., 20.}}	{66.2522, 261.548}	inf	2276.	5979.45	{5696.87, 6332.38}	162.718	0.419152	newly	4341.	2867.78	{2757., 2994.}	33.9373	0.180276	Reff	1.7106	2.72596	{2.7306, 2.75536}	59.3571	ar	73 232.	97 502.	{96 754., 98 216.}	33.1412
43	R0	{2.83096, 16.7077}	{1.90787, 20.}	{{1.89993, 20.}, {1.9121, 20.}}	{32.607, 19.7053}	inf	8628.	4068.7	{4017.27, 4096.71}	52.8431	0.326467	newly	4809.	1624.37	{1573., 1675.}	66.2223	0.471504	Reff	2.43862	1.76045	{1.75335, 1.76646}	27.8095	ar	92 740.	82 165.5	{81 791., 82 576.}	11.4024
44	R0	{2.73678, 11.6021}	{3., 20.}	{{3., 20.}, {3., 20.}}	{9.61787, 72.3826}	inf	6552.	8754.35	{8521.58, 8934.66}	33.6134	0.125751	newly	6185.	4062.92	{3921., 4201.}	34.3101	0.182636	Reff	2.44682	2.63726	{2.62896, 2.64614}	7.78316	ar	92 003.	97 750.1	{97 527., 97 945.}	6.24661
45	R0	{2.14235, 6.98601}	{2.8155, 20.}	{{2.79437, 20.}, {2.84572, 20.}}	{31.4211, 186.286}	inf	2850.	5414.46	{5328.23, 5505.97}	89.9811	0.278639	newly	4808.	2524.43	{2454., 2607.}	47.4952	0.27997	Reff	2.02463	2.60745	{2.5889, 2.64054}	28.7867	ar	82 974.	96 586.8	{96 354., 96 822.}	16.4061
46	R0	{2.23409, 9.21289}	{2.72869, 20.}	{{2.69802, 20.}, {2.77071, 20.}}	{22.139, 117.087}	inf	2544.	4754.44	{4514.07, 5102.92}	86.8883	0.270994	newly	3017.	2192.11	{2023., 2348.}	27.3414	0.139405	Reff	2.12542	2.55312	{2.51805, 2.59251}	20.123	ar	85 163.	95 949.2	{95 541., 96 375.}	12.6654
47	R0	{2.12258, 8.15265}	{2.99173, 19.7745}	{{3., 19.8205}, {3., 20.}}	{40.9478, 142.553}	inf	2945.	6198.43	{5725.46, 6685.25}	110.473	0.322028	newly	3839.	2976.44	{2781., 3202.}	22.4684	0.111165	Reff	1.99728	2.7156	{2.7049, 2.7691}	35.9644	ar	82 690.	97 959.4	{97 158., 98 688.}	18.4659
48	R0	{2.71771, 10.4636}	{2.73229, 19.812}	{{2.48103, 20.}, {3., 20.}}	{7.15002, 89.3424}	inf	2061.	2520.6	{1924.26, 3296.47}	24.7043	0.0907875	newly	2437.	1200.36	{930., 1603.}	50.7444	0.316984	Reff	2.62862	2.62494	{2.41296, 2.87902}	6.96676	ar	91 716.	93 566.4	{91 391., 95 935.}	2.93911
49	R0	{2.44369, 6.66477}	{2.74188, 15.9042}	{{2.09454, 12.9115}, {3., 19.2959}}	{19.1741, 138.631}	inf	1213.	1798.22	{1143.74, 2410.47}	52.0087	0.174615	newly	3032.	1161.94	{787., 1673.}	61.6774	0.435184	Reff	2.39345	2.60157	{2.03568, 2.92872}	15.8918	ar	88 827.	92 737.9	{85 001., 96 446.}	7.21576
50	R0	{2.77383, 17.9775}	{2.16251, 20.}	{{2.15902, 20.}, {2.16482, 20.}}	{22.0388, 11.2502}	inf	14 883.	9337.13	{9282.39, 9379.99}	37.2631	0.20248	newly	6948.	3756.07	{3692., 3829.}	45.9403	0.267167	Reff	2.0682	1.8272	{1.8233, 1.83058}	11.6526	ar	92 334.	91 488.3	{91 028., 91 871.}	0.915881
